# The impact of guidance on the supply of codeine-containing products on their use in intentional drug overdose

**DOI:** 10.1093/eurpub/ckab082

**Published:** 2021-05-26

**Authors:** Emma Birchall, Ivan J Perry, Paul Corcoran, Caroline Daly, Eve Griffin

**Affiliations:** 1 School of Medicine, University College Cork, Cork, Ireland; 2 School of Public Health, University College Cork, Cork, Ireland; 3 National Suicide Research Foundation, Cork, Ireland

## Abstract

**Background:**

Concerns about the misuse of codeine led to the introduction of guidance restricting the supply of over-the-counter (OTC) codeine-containing products in Ireland in 2010. The aim of this study was to examine the impact of this guidance on the national rate of hospital-presenting self-harm involving codeine-related intentional drug overdose (IDO).

**Methods:**

Presentations involving IDO to Irish general hospitals between 1 January 2007 and 31 December 2013, as recorded by the National Self-Harm Registry Ireland, were analyzed. Event-based rates per 100 000 were calculated using national population data. Poisson regression models were used to assess rate changes between pre- and post-guidance periods and to calculate excess presentations.

**Results:**

Between January 2007 and December 2013, a total of 57 759 IDOs were recorded, with 4789 (8.3%) involving a codeine-containing product. The rate of codeine-related IDOs was 20% lower in the period following implementation of the guidance (incidence rate ratio: 0.80; 95% CI: 0.75 to 0.85), representing a total of 509 (95% CI: −624, −387) fewer codeine-related IDOs in that period. Reductions were observed across all ages and were more pronounced for females (0.76, 0.71 to 0.82) than males (0.87, 0.79 to 0.97). The rate of IDOs involving other drugs decreased by 3% in the same period (0.97, 0.95 to 0.98).

**Conclusion:**

Our findings indicate that the rate of codeine-related IDOs was significantly lower in the period following the implementation of the guidance. There is a large body of evidence supporting the restriction of potentially harmful medication as an effective strategy in suicide prevention.

## Introduction

Those who present to hospital with self-harm are at high risk of suicide.^1^ The annual incidence rate of hospital-presenting self-harm is ∼200 per 100 000, with the most common method involved being intentional drug overdose (IDO), present in between 62% and 84% of all presentations.^2–4^ IDO is most common among females and persons aged below 45 years.^5^ Over-the-counter (OTC) medications such as analgesics and opiates are more often consumed in IDOs by young people, whereas prescribed medication, mainly psychotropic drugs such as benzodiazepines, are more common among older people.^5^ Recent studies have found that the incidence of IDOs with OTC medications such as paracetamol and ibuprofen have increased significantly since the 2000s in several countries.^6–9^

Codeine phosphate is a mild-to-moderate opiate analgesic widely used as an OTC pain and cough medication as well as a prescribed medicine.^10^ In recent years, codeine misuse has been of increasing concern, with codeine poisonings among the top five substances most frequently referred to the National Poisonings Information Service in the UK.^11^ A European study estimated that 640 codeine prescriptions are dispensed per 1000 patients,^12^ and a survey of codeine use in Ireland found that 6% of people purchase codeine products on a weekly basis.^13^ Previous research carried out in both the USA^14^^,^^15^ and Australia^16^ have determined that suicide and self-harm rates from intentional drug poisonings involving opiates have been steadily increasing since the early 2000s, with one study concluding that codeine containing medications and their availability have significantly contributed to this increase.^16^ Opiates have also been increasingly linked with deaths in Ireland and internationally.^17^^,^^18^ A recent case fatality study of intentional overdose found that opiate drugs were associated with a 12-fold increased risk of death.^19^

Restricting access to means is an effective strategy in reducing the incidence of self-harm or suicide.^20^ Existing research indicates that the frequency of and trends in drugs used in IDOs reflects their availability and prescribing in a population.^5^ Previous research has shown that measures which limit access to certain medications, via restrictions or withdrawals from the market, can be effective in reducing their use in IDO.^21–23^

In response to the increasing misuse of codeine in IDO, a number of countries have introduced regulations to restrict the sale of OTC codeine containing medication.^24^^,^^25^ Since 1977, codeine has been listed as a controlled drug in The Misuse of Drugs Act in Ireland.^26^^,^^27^ The act currently categorizes drug substances into five schedules, ranging from the most tightly controlled in Schedule 1 to the least tightly controlled in Schedule 5.^28^ Under this, codeine is classed as a Schedule 5 controlled drug, meaning that it is available OTC in pharmacies, but only under the supervision of a pharmacist. In 2010, the Pharmaceutical Society of Ireland (PSI), in conjunction with the Irish Medical Council, issued guidance for pharmacists which framed this previous legislation in relation to the supply of codeine containing products.^29^ The guidance identified specific criteria to be adhered to when selling codeine products, including: the storage of codeine-containing products outside of public view, the provision of only short-term prescriptions, the need to advise patients of the correct usage and risks associated with codeine and the requirement that ‘combination’ products are only dispensed as ‘second-line treatment’. The guidelines were published in May 2010 and inspections of pharmacies commenced from August 2010.

The impact of these restrictions and similar ones in other countries has not been extensively evaluated. A recent study examining the impact of the 2010 Irish guidance regarding codeine sales reported a 33% reduction in cases referred to a national poisons information centre following the 2010 guidance.^30^ In Australia, similar up-scheduling of codeine compound analgesics in 2010 did not impact on an increasing trend in codeine-related poison centre calls, while more recent legislation moving codeine-related medication to prescription only resulted in fewer calls recorded.^31^^,^^32^ We sought to investigate if the guidance introduced in Ireland in 2010 impacted on the rate of codeine-related IDOs, using national surveillance data on hospital-presenting self-harm.

## Methods

### Data sources

The National Self-Harm Registry Ireland records information on presentations to every hospital emergency department in the Republic of Ireland (both adult and paediatric) as a result of self-harm. All presentations to hospital involving IDOs recorded by the Registry between 1 January 2007 and 31 December 2013 were included in this study.

The Registry uses the following as its definition of self-harm: ‘an act with non-fatal outcome in which an individual deliberately initiates a non-habitual behaviour that without intervention from others will cause self-harm, or deliberately ingests a substance in excess of the prescribed or generally recognized therapeutic dosage, and which is aimed at realizing changes that the person desires via the actual or expected physical consequences’.^33^ Data are gathered by trained data registration officers who work independently of the hospitals and follow the Registry's standardized methods of case ascertainment. The validity of the data and consistency of case ascertainment over time is ensured by regular training of data registration officers, reinforcement of inclusion and exclusion criteria, low staff turnover and annual quality control exercises. More detailed standard operating procedures of the Registry have been previously described.^34^

The Registry records up to a maximum of five methods per self-harm presentation, using International Statistical Classification of Diseases and Related Health Problems 10th Revision (ICD-10). Intentional drug overdose (IDO) presentations are identified by codes X60–X64. Intentional self-poisoning using other substances (X65–X69) are not included in this study. The names of individual drugs taken in IDO are recorded by the Registry and these names were used to construct the drug categories included in the analysis. In 1.9% of IDOs, the drug names were recorded as unknown.

Population data from the 2011 national census and annual population estimates for intercensal years (disaggregated by sex and age group) were obtained from the website of the Irish Central Statistics Office (http://www.cso.ie/) and used to calculate event-based rates of codeine-related IDOs.

### Study design

In line with the introduction of the guidance, we established a pre-guidance period of 1 January 2007–31 March 2010 and a post-guidance period of 1 October 2010–31 December 2013 in order to determine the impact of the guidance on hospital-presenting IDO. The period 1 April to 30 September 2010 (quarter 2 and 3) was excluded from all analyses, as this was deemed to be the ‘bedding-in’ period for pharmacists to fully implement the new guidelines. The primary outcome measure for this study was the event-based rate per 100 000 of codeine-related IDO. Control measures consisted of the rate of IDOs involving other drugs, as well as IDOs involving non-opioid analgesics and other opiates.

### Statistical analyses

The overall incidence of hospital-presenting IDO and codeine-related IDO were calculated based on the number of IDO presentations made to hospital in each calendar year. In the pre-post analysis, quarterly rates per 100 000 population were calculated for all outcome measures. We calculated 95% confidence intervals (CIs) for these rates, using the normal approximation for the Poisson distribution. Rates per 100 000 were also constructed according to gender and age. Initially, changes in the rate of IDOs were examined across five-year age bands. Due to small numbers and following an inspection of the trends in these groups, three larger age groups were used in the final analysis: <25, 25–64 and 65 years and over.

Poisson regression analyses were used to assess the rate changes between the pre- and post-guidance periods and also to calculate the quarterly percentage change (QPC) in rates for each time period, with incidence rate ratios (IRRs) and their 95% CIs being reported. Separate models were constructed to examine changes according to gender and age.

The IRR values, together with the observed number of presentations, were used to estimate the expected and the excess number of codeine-related IDOs that occurred in the post-guidance period, compared with the pre-guidance period. The number of expected presentations was calculated by dividing the obtained IRR by the observed number of presentations in this post-guidance period. The excess number of codeine-related IDOs was calculated by multiplying the expected number of IDOs by the IRR value minus one. The 95% CI of the IRR was used to estimate the 95% CI of the excess number of codeine-related IDOs. The latter’s lower confidence limit minus the expected number of paracetamol-related IDOs and the equivalent calculations were made to generate the upper confidence limit of the excess number. Analyses were conducted using Microsoft Excel, SPSS Version 25 and Stata IC Version 12.0.

### Ethical approval

Ethical approval for the Registry has been granted by the National Research Ethics Committee of the Faculty of Public Health Medicine and the Cork Research Ethics Committee. This study is reported in accordance with the Strengthening the Reporting of Observational Studies in Epidemiology (STROBE) guidelines for observational studies.^35^

## Results

### Sample characteristics

Between 1 January 2007 and 31 December 2013, there were a total of 57 759 [annual mean (range): 8251 (7456–8757)] presentations to hospital as a result of IDO, representing 70.4% of all presentations due to self-harm. The majority of these presentations were by females (*n* = 33 738; 56.8%) and those aged between 25 and 44 years (*n* = 35 198; 60.9%). Opiates were involved in 12.9% (*n* = 7434) of all IDOs. At least one codeine-containing medication was recorded in 8.3% (4789) of these. The annual mean number of codeine-related IDOs was 684 (range: 545–774).

### Rates of IDO

The total, male and female rates of hospital-presenting IDO were 183.7 (95% CI: 182.3 to 185.3), 153.8 (95% CI: 151.9 to 155.8) and 213.3 (95% CI: 211.0 to 215.6) per 100 000, respectively. The female rate was significantly higher than the male rate (IRR: 1.39; 95% CI: 1.36 to 1.41). The total, male and female rates of codeine-related IDOs for the same period were 15.3 (95% CI: 14.7 to 15.5), 9.9 (95% CI: 9.5 to 10.5) and 20.4 (95% CI: 19.5 to 20.9) per 100 000, respectively. Similar to all IDOs, the female codeine-related IDO rate was significantly higher than the male rate (IRR: 2.03; 95% CI: 1.91 to 2.15).

**Figure 1:**
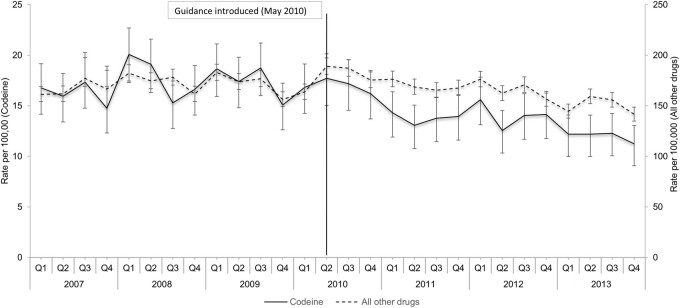
Trends in rates per 100 000 of all IDOs and codeine-related IDOs between 2007 and 2013. Vertical line indicates the quarter [Q2 (May) 2010] when codeine guidance was introduced in Ireland

Figure 1 illustrates the quarterly rates of codeine-related IDO and IDOs involving other drugs across the study period. Considering all IDOs, the rate in 2013 was 11% lower than the rate in 2007 (IRR: 0.89; 95% CI: 0.86 to 0.92). For codeine-related IDO, the rate in 2013 was 25% lower (95% CI: 0.75, 0.67 to 0.83). Between January 2007 and March 2010 (pre-guidance period), there was no significant trend observed in codeine-related IDOs (QPC: +0.1%, 95% CI: −0.9 to +1.2) or those involving other drugs (QPC: −0.1%, −0.4 to +0.3). A pronounced decrease was observed between April 2010 and April 2011 in codeine-related IDOs, where the rate decreased from 17.4 to 12.9 per 100 000. In the second half of the study period, between October 2010 and December 2013, a decreasing trend in the rate of codeine-related IDOs was detected (QPC: −2.0%, −3.1 to −0.8). A similar trend was observed in the same period for rates of IDOs involving other drugs (QPC: −1.5%, −1.8 to −1.1).

### Change in codeine-related IDO after introduction of guidance

Considering the pre- and post-guidance periods of January 2007 to March 2010 (pre) and October 2010 to December 2013 (post), the rate of codeine-related IDO was 20% lower in the post-guidance period (IRR: 0.80; 95% CI: 0.75 to 0.85), translating to 509 fewer presentations than expected (−624, −387) (tables 1 and 2). The rate of IDOs involving other drugs was also lower in the post-guidance period, although the magnitude of change was much less pronounced (0.97, 0.95 to 0.98). An 11% reduction in the rate of IDOs involving non-opioid analgesics was observed (0.89, 0.87 to 0.93), while the rate of IDOs involving other opiates was not significantly different in the post-guidance period (0.98, 0.91 to 1.06).

**Table 1:** Change in rate per 100 000 of IDOs involving codeine, all other drugs (excluding codeine), analgesics and other opiates pre- and post-guidance

	Pre-guidance		Post-guidance		
	January 2007 to March 2010		October 2010 to December 2013		
	*n*	Rate per 100 000 (95% CI)	*n*	Rate per 100 000 (95% CI)	IRR (95% CI)
Codeine	2452	17.1 (16.5 to 17.8)	2009	13.5 (12.9 to 14.1)	0.80 (0.75 to 0.85)
All other drugs	24 419	170.9 (168.7 to 173.0)	24 284	163.2 (161.1 to 165.2)	0.97 (0.95 to 0.98)
Non-opioid analgesics	9292	64.9 (63.4 to 66.3)	8586	57.7 (56.5 to 58.9)	0.90 (0.87 to 0.93)
Other opiates	1273	8.9 (8.4 to 9.4)	1286	8.6 (8.2 to 9.1)	0.98 (0.91 to 1.06)

**Table 2:** Change in rate of codeine-related IDO according to age and gender

	Pre-guidance		Post-guidance		Change		
	January 2007 to March 2010		October 2010 to December 2013				
	n	Rate per 100 000 (95% CI)	n	Rate per 100 000 (95% CI)	IRR (95% CI)	Expected	Excess (95% CI)
All							
All ages	2452	16.9 (16.2 to 17.6)	2009	13.5 (12.9 to 14.1)	0.80 (0.75 to 0.85)	2518	−509 (−624, −387)
<25 years	894	16.5 (15.5 to 17.7)	646	12.8 (11.8 to 13.8)	0.77 (0.70 to 0.85)	837	−191 (−253, −122)
25–64 years	1507	19.2 (18.3 to 20.2)	1331	16.5 (15.6 to 17.4)	0.86 (0.80 to 0.92)	1553	−222 (−316, −120)
65+ years	51	3.2 (2.5 to 4.2)	32	1.8 (1.3 to 2.5)	0.56 (0.36 to 0.87)	57	−25 (−37, −8)
Male							
All ages	768	10.6 (9.9 to 11.4)	683	9.2 (8.6 to 9.9)	0.87 (0.79 to 0.97)	783	−100 (−167, −26)
<25 years	243	9.4 (8.3 to 10.6)	172	6.7 (5.8 to 7.8)	0.71 (0.59 to 0.87)	241	−69 (−100, −32)
25–64 years	510	12.9 (11.8 to 14.1)	498	12.3 (11.3 to 13.4)	0.95 (0.84 to 1.08)	523	−25 (−83, 41)
65+ years	15	2.1 (1.3 to 3.5)	13	1.6 (1.4 to 2.8)	0.76 (0.36 to 1.59)	17	−4 (−11, 10)
Female							
All ages	1685	23.1 (22.1 to 24.3)	1326	17.6 (16.7 to 18.6)	0.76 (0.71 to 0.82)	1741	−415 (−507, −316)
<25 years	651	25.9 (23.9 to 27.9)	474	19.1 (17.4 to 20.9)	0.74 (0.65 to 0.83)	643	−169 (−222, −110)
25–64 years	998	25.6 (24.1 to 27.3)	833	20.5 (19.1 to 21.9)	0.80 (0.73 to 0.88)	1043	−210 (−284, −130)
65+ years	36	4.1 (2.9 to 5.7)	19	1.9 (1.3 to 3.1)	0.48 (0.27 to 0.83)	40	−21 (−29, −7)

The changes in codeine-related IDOs were further examined according to age and gender (table 2). Considering gender, the reduction in the rate of codeine-related IDOs was more pronounced among females (−24%; 0.76, 0.71 to 0.82) than in males (−13%, 0.87, 0.79 to 0.97) representing 415 (−507, −316) and 100 (−167, −26) fewer presentations than expected. The overall reduction in codeine-related IDOs in the post-guidance period was reflected across all age groups, −23% (0.77, 0.70 to 0.85) in those aged under 25 years, −14% in those aged 25–64 years (0.86, 0.80 to 0.92) and −44% (0.56, 0.36 to 0.87) among those aged 65 years and older. The decrease among young people aged below 25 years was similar for males (0.71, 0.59 to 0.87) and females (0.74, 0.65 to 0.83). However, the observed decreases among 25–64 year olds and those aged 65 years and over were significant for females only [25–64 years: 0.80, (0.73 to 0.88) and 65 years and over: 0.48, (0.27 to 0.83)].

## Discussion

The aim of this study was to examine the impact of guidance introduced by the Pharmaceutical Society of Ireland in 2010 on the rate of codeine related IDOs. We found that in the post-guidance period (October 2010 to December 2013), the rate of codeine-related IDOs was 20% lower than the pre-guidance period (January 2007 to March 2010), a total of 509 fewer presentations in the post-guidance period than would be expected. The reduction was most pronounced among females and among those aged below 25 years. The reduction observed was most apparent between April 2010 and April 2011, and the rate of codeine-related IDOs continued to decrease (−2% per quarter) in the post-guidance period, suggesting that the introduction of the guidance had an immediate impact on the use of codeine products in self-harm. The rate of IDOs involving other drugs as well as IDOs involving non-opioid analgesics were also lower in the post-guidance period, although the reductions were not as pronounced. In addition, a similar decreasing trend in the rate of IDOs involving other drugs in the period following the guidance was also observed.

Our findings are consistent with another Irish study, which reported a 33% reduction in the number of reported codeine poisonings to a national poisoning centre in the year following the introduction of the guidance.^30^ Similarly, studies which have examined the impact of guidance regulating codeine use among children have also shown similar decreases.^36^^,^^37^ In contrast, an Australian study found that similar re-scheduling did not impact on calls to a national poisoning centre,^31^ while a more resent re-scheduling of codeine products to ‘prescription only’ resulted in a significant drop in calls and a decrease in codeine sales.^32^ However direct comparisons cannot be made as these calls to poisoning centres do not represent overdoses cases directly, rather more generally concerns regarding medications. While is difficult to identify via observational studies the specific components of these guidelines which led to these reductions, this consultation with a pharmacist which would involve education about the side-effects and the dangers of taking codeine-containing drugs may have been an integral component of the guidance in explaining the impact.^28^ Future research could seek to evaluate the effectiveness of specific components of such regulations.

Unlike previous studies, this study further examined the impact of implemented guidance on codeine-related IDOs according to age and gender. While the rates of female codeine-related IDOs were higher overall, the reduction in these presentations was also greater among females, following the introduction of the guidance. While we found that the rate of codeine-related IDOs decreased across all age groups, this reduction was most pronounced for young people aged below 25 years. However, those aged between 25 and 64 years were at highest risk of using a codeine-containing medication in IDOs and perhaps most significantly, accounted for a larger percentage in the post-guidance period (66.3%) when compared with the pre-guidance period (61.5%). This provides evidence to support previous research which also concluded that young to middle-aged people tend to be most likely to self-harm using IDO.^13–15^ For this reason, they have been identified as a target group for suicide prevention,^20^ making research into the effective prevention of self-harm in this group timely and necessary in order to further inform intervention and prevention strategies.

It is challenging to evaluate the impact of regulations such as these due to inherent methodological issues with observational studies—including risk of bias and confounding—as well as a lack of high-quality national data.^38^ This is reflected in the relatively small number of published studies from other countries. We used several approaches to strengthen our study. We used national surveillance data from a national self-harm registry, which is one of the few national surveillance systems monitoring the incidence of hospital-presenting self-harm. This approach ensures that all hospital-treated cases of IDO are included in this study and overcomes some of the limitations of poisons information centre data, including difficulties in establishing intent. Furthermore, we used several approaches to validate that the changes observed were linked with the exposure we examined. This included the construction of equal pre- and post-guidance periods, as well as the exclusion of a ‘bedding-in’ period where the guidance was being rolled out and implemented. We also used a number of control measures to compare the changes observed to other IDO presentations and see if there was a substitution effect among medications, of which there was none.

However, there were some limitations to the study. Firstly, hospital-presenting self-harm was examined, and therefore IDOs which were untreated or seen only by a general practitioner were not included in this study. Lastly, there may have been other legislation or social and economic factors impacting the rates of IDOs during this period that were not fully accounted for in this study. In order to accurately estimate the impact of the guidance, we used the rate of other IDOs and those involving non-opioid analgesics and other opiates as control measures for the changes in both time periods. This also served to identify any substitution effects in the types of drugs used. The decrease in rates of IDO involving other drugs was much less pronounced, while rates of IDOs involving other opiates did not change. However it is difficult to be conclusive about whether the patterns observed were as a direct result of the regulations introduced, in particular as a similar decreasing trend in the post-guidance period was observed for IDOs involving other drugs. For example, the pre-guidance period we used included the onset of the global economic recession in 2008, which was associated with increases in both the incidence of self-harm and suicide in Ireland.^39^ The highest rates of IDOs involving other drugs and codeine-related IDOs were observed during the first quarter of 2008. However, this previous study did show that the rates of self-harm continued to rise during the period of austerity measures in Ireland following the recession between 2009 and 2012. The lower rates of IDO in this period could be due to an increased use in more lethal methods of self-harm during this time, as has been shown in young people.^2^ However, the reduction in codeine-related IDOs was greater than for the other drug groups examined, suggesting that the regulations were effective in reducing the use of codeine in IDO.

Legislation limiting the sale of other medications such as reducing paracetamol pack-sizes and the withdrawal of drugs such as distalgesic^21^^,^^22^ have also been shown to be effective in reducing the use of these drugs in IDO. This study further adds to this evidence base that universal suicide prevention measures can have positive impacts on those at risk of self-harm and suicide and demonstrates the important role that means restrictions measures have in national and international suicide prevention strategies and policy.^20^^,^^40^ This study has indicated that the introduction of guidance by the PSI in May 2010 which restricted the supply of codeine-containing drugs was associated with a significant reduction in codeine-related IDOs in period following its introduction. This study will inform policy makers and future governments on the effectiveness of means restriction interventions to prevent suicidal behaviour.
